# The ABC toxin complex from *Yersinia entomophaga* can package three different cytotoxic components expressed from distinct genetic loci in an unfolded state: the structures of both shell and cargo

**DOI:** 10.1107/S2052252524001969

**Published:** 2024-05-01

**Authors:** Jason N. Busby, Sarah Trevelyan, Cassandra L. Pegg, Edward D. Kerr, Benjamin L. Schulz, Irene Chassagnon, Michael J. Landsberg, Mitchell K. Weston, Mark R. H. Hurst, J. Shaun Lott

**Affiliations:** a School of Biological Sciences, University of Auckland, Auckland 1142, New Zealand; b School of Chemistry and Molecular Biosciences, University of Central Queensland, Brisbane, Queensland 4072, Australia; c Resilient Agriculture, AgResearch, Lincoln Research Centre, Christchurch 8140, New Zealand; University of Hamburg, Germany

**Keywords:** *Yersinia entomophaga*, ABC toxins, macromolecular machines, protein structures, multi-protein complexes

## Abstract

Bacterial ABC toxin complexes (Tcs) comprise three core proteins: TcA, TcB and TcC. The TcA protein forms a pentameric assembly that attaches to the surface of target cells and penetrates the cell membrane. The TcB and TcC proteins assemble as a heterodimeric TcB–TcC subcomplex that makes a hollow shell. This TcB–TcC subcomplex self-cleaves and encapsulates within the shell a cytotoxic ‘cargo’ encoded by the C-terminal region of the TcC protein. Here, we describe the structure of a previously uncharacterized TcC protein from *Yersinia entomophaga*, encoded by a gene at a distant genomic location from the genes encoding the rest of the toxin complex, in complex with the TcB protein. When encapsulated within the TcB–TcC shell, the C-terminal toxin adopts an unfolded and disordered state, with limited areas of local order stabilized by the chaperone-like inner surface of the shell. We also determined the structure of the toxin cargo alone and show that when not encapsulated within the shell, it adopts an ADP-ribosyltransferase fold most similar to the catalytic domain of the SpvB toxin from *Salmonella typhimurium*. Our structural analysis points to a likely mechanism whereby the toxin acts directly on actin, modifying it in a way that prevents normal polymerization.

## Abstract 2

A cytotoxin encoded by an ‘orphan’ genetic locus in the insect pathogen *Yersinia entomophaga* is shown to be a functional part of an ABC toxin complex, and the structures of both the RHS-repeat-containing ‘shell’ and the free toxin alone are determined by X-ray crystallography. The structure of the toxin indicates that it most likely functions by directly modifying actin in the target cell.

## 1. Introduction

The ABC toxins are a class of multi-subunit toxin complexes present in several entomopathogenic bacteria that have three main protein components: TcA, TcB and TcC (ffrench-Constant & Waterfield, 2006 ▸). The structures of several ABC toxin complexes have been determined, and these structures have enabled the mechanism of action of this class of toxin to be elucidated (Landsberg *et al.*, 2011 ▸; Busby *et al.*, 2013 ▸; Gatsogiannis *et al.*, 2013 ▸, 2016 ▸, 2018 ▸; Meusch *et al.*, 2014 ▸; Piper *et al.*, 2019 ▸; Leidreiter *et al.*, 2019 ▸; Roderer *et al.*, 2019*a* ▸). ABC toxins exist in two sub-types: type I, where five copies of a single TcA protein form a pentameric assembly that acts as a nano-injection device to penetrate the cell membrane of target cells and deliver the toxic payload (Gatsogiannis *et al.*, 2013 ▸); and type II, where the TcA protein is split into two halves, transcribed from separate genes, and in some cases also incorporate other accessory proteins (Landsberg *et al.*, 2011 ▸; Piper *et al.*, 2019 ▸). In both cases, the pentamer is topped with a single copy of each of the TcB and TcC proteins that come together to form a hollow shell, encapsulating the cytotoxic C-terminal domain of TcC (TcC^CTD^) as ‘cargo’ inside the shell (Busby *et al.*, 2013 ▸; Meusch *et al.*, 2014 ▸).

The hollow shell formed by TcB and TcC consists primarily of repeated copies of the rearrangement hot-spot (RHS) repeat sequence, also described as ‘YD’ repeats because they contain a highly conserved tyrosine–aspartate dipeptide motif. RHS repeats are found in many different bacterial species, both in ABC toxin complexes and in other genes involved in pathogenesis or inter-strain competition (Poole *et al.*, 2011 ▸; Koskiniemi *et al.*, 2013 ▸; Jurėnas *et al.*, 2021 ▸; Günther *et al.*, 2022 ▸), and are also found in the teneurins, which play a key role in inter-cellular communication in eukaryotes (Ferralli *et al.*, 2018 ▸; Jackson *et al.*, 2018 ▸, 2019 ▸; Li *et al.*, 2018 ▸; Araç & Li, 2019 ▸; del Toro *et al.*, 2020 ▸; Meijer *et al.*, 2022 ▸).

Bacterial RHS-repeat-containing proteins belong to a broad family of proteins known as polymorphic toxin systems (Zhang *et al.*, 2012 ▸; Jamet & Nassif, 2015 ▸). Polymorphic toxins are a class of bacterial toxin delivery system with a characteristic genomic layout, usually consisting of a signal peptide or secretory domain, multiple copies of a repeating unit, a releasing peptidase, and a highly variable C-terminal toxin domain. RHS cassettes can be swapped with other orphaned cassettes in the genome by recombination, allowing for a variety of different toxin cargoes to share a common delivery vehicle. Due to the high degree of variation in their sequences, the toxin cargoes have also been described as ‘hypervariable regions’, or HVRs (Roderer & Raunser, 2019 ▸).

The bacterium *Yersinia entomophaga* is pathogenic to a variety of insect species (Hurst *et al.*, 2011*a* ▸). One of the primary determinants of its pathogenicity is a type II ABC toxin complex, Yen–Tc, encoded by two *tcA* genes (each encoding half of the TcA component); a single *tcB* gene; two distinct *tcC* genes, each encoding different C-terminal toxin domains; and two accessory chitinases (Hurst *et al.*, 2011*b* ▸). On the basis of their amino acid sequences, the two TcC toxins are predicted to be homologous to cytotoxic necrotizing factors (that constitutively activate Rho GTPase) and nucleotide deaminases, respectively (Busby *et al.*, 2013 ▸).

The genome sequence of *Y. entomophaga* (Hurst *et al.*, 2016 ▸) contains several genes encoding RHS-repeat-containing proteins at different genomic locations. The *rhs2* region, located ∼900 000 base-pairs from the Yen–Tc-encoding gene cluster in the genome, contains a gene (PL78_18780) that shows remarkable sequence similarity to the other *tcC*-type genes. An amino acid sequence alignment (Sievers *et al.*, 2011 ▸) of YenC1, YenC2 and PL78_18780 shows a high level of similarity within the N-terminal domain (≥60% pairwise sequence identity over ∼680 residues), but the C-terminal domains show no detectable sequence similarity (see the supporting information), consistent with the presence of a hypervariable region. ‘Orphaned toxin cassettes’ have been described for other polymorphic toxin systems (Koskiniemi *et al.*, 2014 ▸) and so we speculated that PL78_18780, which has been re-annotated as *yenC3* (Paulson *et al.*, 2021 ▸), may indeed encode a third TcC protein, carrying a different C-terminal cargo, that may in turn form a complex with YenB in a similar manner to YenC1 and YenC2 (Fig. S1 of the supporting information). The *rhs2* locus also encodes other potential virulence effector proteins, including a putative YopT-type cysteine protease and a putative vertebrate C-lysozyme inhibitor (Hurst *et al.*, 2016 ▸), suggesting that this region of the genome is a pathogenicity island required in the infection process. Congruent with this idea, the YenC3 transcript is upregulated during the early infection stage of infection of the wax moth *Galleria mellonella* by *Y. entomophaga* (Paulson *et al.*, 2021 ▸).

We therefore set out to characterize the predicted RHS-repeat-containing protein YenC3 to determine if it is an additional TcC protein associated with Yen–Tc and whether it functions as a toxin.

## 2. Results

### 2.1. Co-expression and purification of YenB and YenC3

To test whether YenC3 could form a complex with YenB, the *yenB* and *yenC3* gene products were co-expressed in *E. coli* from a single expression plasmid. YenB was expressed with an N-terminal His_6_-tag, whereas YenC3 was left untagged. The two proteins formed a complex that bound to immobilized metal affinity chromatography (IMAC) resin, and they remained associated with each other throughout further purification by size-exclusion chromatography (SEC). As with other TcC proteins (Busby *et al.*, 2013 ▸), YenC3 underwent self-cleavage at the interface between its N- and C-terminal domains, with both domains remaining associated with the complex (Fig. S2). The observations that YenC3 is able to form a stable complex with YenB, and that its C-terminal sequence is cleaved but remains associated with the complex are entirely consistent with YenC3 functioning as a *bona fide* TcC protein.

### 2.2. The YenB/YenC3 complex structure

**Figure 1:**
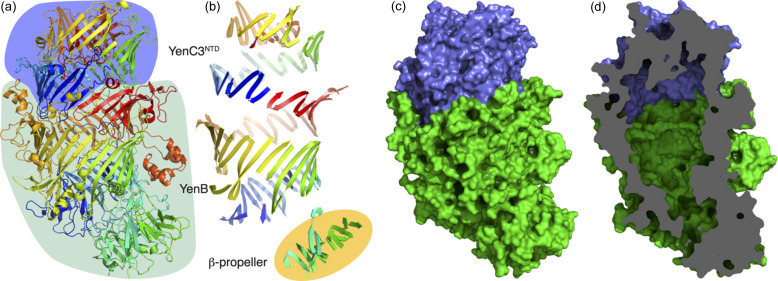
(*a*) Crystal structure of YenB/YenC3^NTD^, coloured from blue at the N-terminus to red at the C-terminus. YenB is highlighted in green and YenC3^NTD^ in blue. (*b*) Strands of the β-sheet shell around the central cavity, coloured as in (*a*). The β-propeller domain is highlighted in orange. Surface representations showing (*c*) the exterior and a vertical slice through showing (*d*) the internal cavity. YenB is shown in green and YenC3^NTD^ is shown in blue.

**Figure 2:**
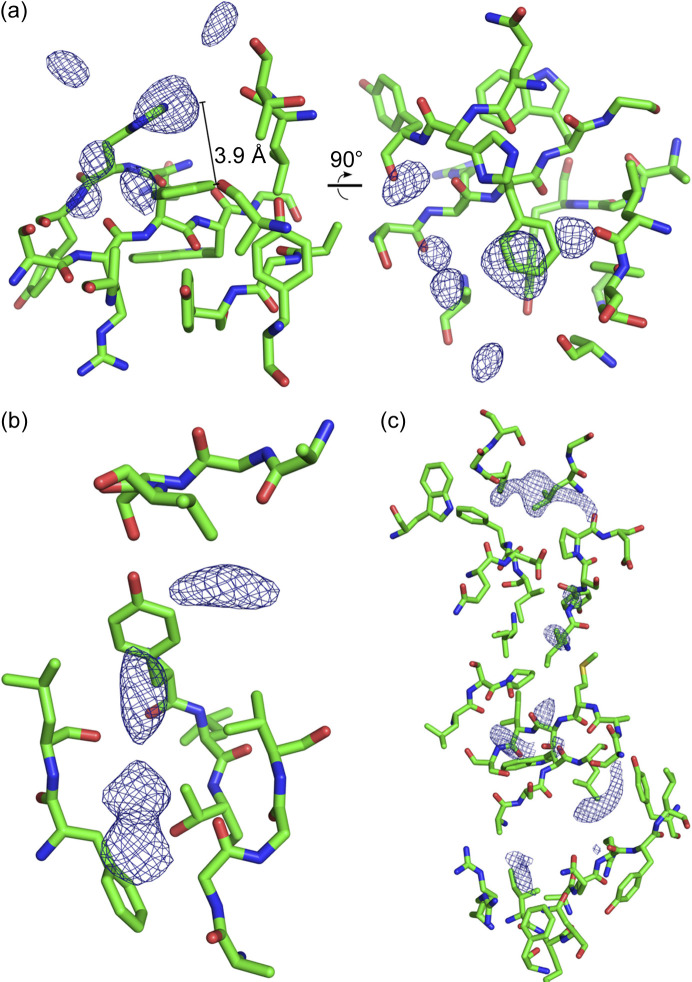
Electron density features on the interior face of the hollow shell. (*a*) Flattened volume of electron density stacking 3.9 Å above the plane of a phenyl­alanine residue. (*b*) and (*c*) Areas of tube-like electron density (*F*_o_ − *F*_c_ map, σ = 3.0) occupying hydro­phobic clefts on the interior surface.

The complex consisting of YenB and both the N- and C-terminal domains of YenC3 (YenC3^NTD^ and YenC3^CTD^) was crystallized, X-ray diffraction data were collected, and the structure was determined by molecular replacement using the structure of YenB/YenC2^NTD^ (PDB entry 4igl; Busby *et al.*, 2013 ▸) as the search model (Table 1 ▸, Fig. 1 ▸). Overall, the structure is extremely similar to the structure of YenB/YenC2^NTD^ which we had determined previously (Busby *et al.*, 2013 ▸, 2016 ▸). An RMSD of 0.77 Å over 2092 superimposed residues was calculated (Krissinel, 2012 ▸), with the major point of difference being that the YenC2^CTD^ had to be removed from the YenB/YenC2 complex before it could be crystallized (Busby *et al.*, 2013 ▸). In contrast, the complete YenB/YenC3 sequence was crystallized, with YenC3^CTD^ still present in the complex. Despite this, no continuous electron density could be observed for YenC3^CTD^. We therefore concluded that the C-terminal domain is most likely encapsulated in an unfolded or disordered state, explaining why it is not visualized in the spatially averaged structure produced by X-ray crystallography (Busby *et al.*, 2013 ▸; Meusch *et al.*, 2014 ▸; Roderer *et al.*, 2019*b* ▸). Close inspection of the electron density delivered further support for this conclusion, with tubes and patches of electron density packing against several regions of the interior surface of the shell observed, which could not be accounted for by the protein residues encoding for the shell itself (Fig. 2 ▸).

In our previous structure of the YenB/YenC2^CTD^ shell, we observed multiple hydro­phobic patches, derived from copies of the RHS repeat, distributed throughout the mostly positively charged interior shell surface. We proposed that this interior surface could stabilize an unfolded, encapsulated cargo protein in a chaperone-like fashion (Busby *et al.*, 2013 ▸; Meusch *et al.*, 2014 ▸). Equivalent hydro­phobic patches are also a feature of the interior YenB/YenC3 shell surface, and are usually found in close proximity to the putative cargo protein density described above, supporting this hypothesis that they stabilize the unfolded cargo. For example, an area of flat electron density was seen ∼3.9 Å from the plane of a phenyl­alanine residue (Y1220), possibly representing aromatic residues of the cargo protein forming π-stacking interactions [Fig. 2 ▸(*a*)]. Multiple examples of tubes of electron density packing into hydro­phobic clefts on the inner surface of the shell were also visible in the structure reported here [Figs. 2 ▸(*b*) and 2 ▸(*c*)]. These observations are consistent with previous reports that the HVR of the TccC3 toxin from *Photorhabdus luminescens* is unfolded within its cognate shell (Gatsogiannis *et al.*, 2018 ▸; Belyy *et al.*, 2022 ▸).

To independently confirm that YenC3^CTD^ was encapsulated within the YenB/YenC3^NTD^ complex, we subjected the YenB/YenC3 complex to small-angle X-ray scattering analysis. The protein sample was purified to homogeneity by SEC (Fig. S2). A concentration series was analysed using SEC with multi-angle light scattering (SEC-MALS) to confirm that the complex formed a 1:1:1 ratio of the expected size, with no aggregation or concentration-dependent oligomerization (Fig. S3). The sample showed no change in elution volume or molecular mass across the concentration range, and the observed molecular mass calculated at the leading edge of the peak was consistent with a 1:1:1 ratio of YenB:YenC3^NTD^:YenC3^CTD^ in the complex.

**Figure 3:**
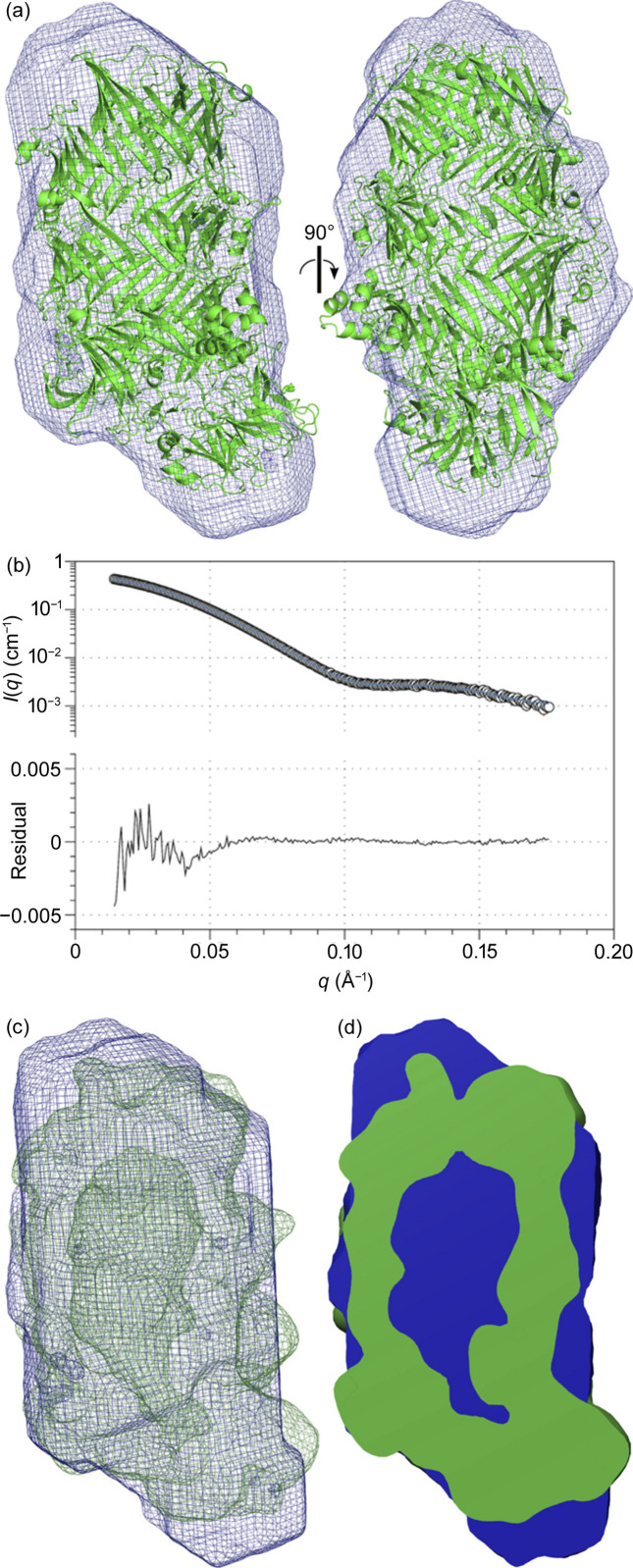
(*a*) Small-angle X-ray scattering bead model of YenB/YenC3^NTD^/YenC3^CTD^ (blue mesh) with the YenB/YenC3^NTD^ crystal structure superimposed. (*b*) Experimental small-angle X-ray scattering of YenB/YenC3^NTD^/YenC3^CTD^ (circles) and the theoretical scattering of the bead model (blue line). The residual plot demonstrates a very good fit of the theoretical scattering to the experimental data: χ^2^ = 0.7548. (*c*) SAXS bead model (blue mesh) with the crystal structure (green mesh) superimposed. (*d*) Vertical slice-through of (*c*) showing the large internal cavity present in the crystal structure (green) but absent in the SAXS model (blue).

Small-angle X-ray scattering data were subsequently collected from this sample (Fig. S4, Table 2 ▸) and an *ab initio* bead model built from the solution scattering data showed similar dimensions to the crystal structure (Fig. 3 ▸), the main difference being the presence of density inside the centre of the hollow ‘shell’. In contrast, bead models built from the YenB/YenC2^NTD^ complex lacking the C2^CTD^ domain show a similar overall shape with a large internal cavity (Busby *et al.*, 2013 ▸). We therefore conclude from the X-ray crystal structure and SAXS analyses that YenC3^CTD^ is encapsulated within the cage in an unfolded state, and further analysis of the SAXS data (see the supporting information) supported this conclusion. This situation is likely to be the case for other TcB–TcC complexes as well.

### 2.3. Determination of the YenC3^CTD^ HVR crystal structure

We hypothesized that the C-terminal HVR domain of YenC3 is most likely a toxin active against insects, as is the case with other characterized TcC proteins, which cause apoptotic cell death in the epithelial cells of the insect midgut by disrupting the actin cytoskeleton (Hurst *et al.*, 2011*b* ▸; Aktories *et al.*, 2015 ▸). The amino acid sequence of YenC3^CTD^ shows two distinct regions. Immediately downstream of the self-cleavage site are four copies of a proline-rich repeat with the consensus sequence PPPPPPMMGGN, which are likely to form extended poly proline helical segments (Adzhubei *et al.*, 2013 ▸). These repeats are followed by a predicted globular domain of ∼230 amino acids. A BLAST search of the Genbank non-redundant protein database with the full YenC3^CTD^ HVR sequence identified a small family of RHS-repeat-containing proteins, with orthologues in a range of Gram-negative bacterial species, including *Morganella morganii*, *Vibrio parahaemolyticus* and several *Pseudomonas* species. None of the identified orthologues are functionally or structurally characterized.

**Figure 4:**
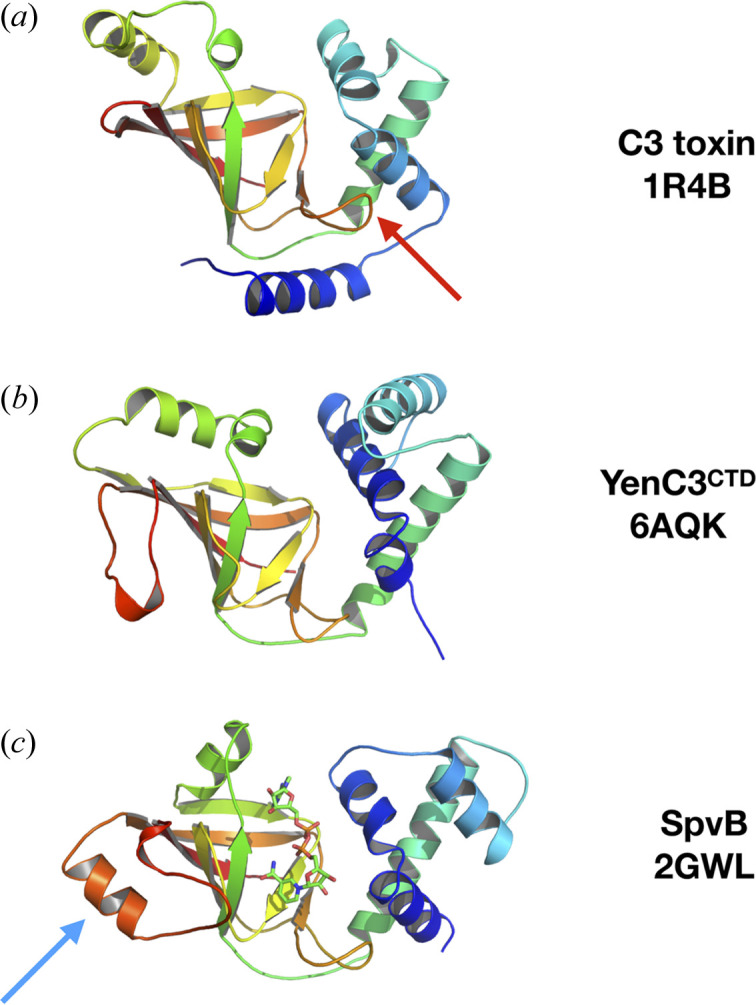
(*a*) The C3 exoenzyme from Clostridium botulinum (PDB entry 1r4b). The substrate recognition loop is indicated with a red arrow. (*b*) The crystal structure of YenC3CTD (PDB entry 6aqk). (*c*) The structure of SpvB from *S. typhimurium* in complex with NAD (shown as sticks) (PDB entry 2gwl). The helical insertion in the central β-sheet is indicated with a blue arrow. All structures are coloured from blue at the N-terminus to red at the C-terminus.

In order to better understand the function of the HVR of YenC3, we expressed, purified and crystallized YenC3^CTD^, excluding the poly proline repeat region, as extended proline-rich sequences tend to crystallize poorly (Williamson, 1994 ▸). We determined its crystal structure (PDB entry 6aqk) using anomalous diffraction from a platinum derivative (Fig. 4 ▸, Table 1 ▸).

### 2.4. Analysis of the YenC3^CTD^ structure

A *DALI* (Holm, 2019 ▸) search of the PDB revealed that the structure of YenC3^CTD^ is most similar to a structurally conserved family of mono-ADP-ribosyltransferase toxins (CATH Superfamily 3.90.176.10). The closest structural homologues are the catalytic domain of the *Salmonella typhimurium* virulence protein SpvB (PDB entry 2gwl; Margarit *et al.*, 2006 ▸), which ADP-ribosylates actin (Margarit *et al.*, 2006 ▸); and the C3 secreted toxin from *Clostridium botulinum* (PDB entry 1r4b; Margarit *et al.*, 2006 ▸), which ADP-ribosylates Rho GTPases (Aktories & Frevert, 1987 ▸; Ménétrey *et al.*, 2008 ▸). The core ADP-ribosyltransferase folds of YenC3^CTD^, SpvB and C3 toxin are all very similar, with an N-terminal helical domain separated from a β-sheet domain by the NAD-binding active site. Consistent with this structural similarity, the inferred active site cleft of YenC3^CTD^ contains the key catalytic arginine, serine and glutamate residues (R839, S881 and E919) that are conserved in the cholera toxin (CT) group of ADP-ribosyltransferases (Simon *et al.*, 2014 ▸).

The toxic HVR regions of the well characterized ABC toxins from *P. luminescens* are also ADP-ribosyltransferases. Like the *C. botulinum* C3 toxin, the HVR of TccC5 from *P. luminescens* ADP-ribosylates Rho proteins, causing constitutive activation and dysregulation of the actin cytoskeleton (Lang *et al.*, 2010 ▸). In contrast, the HVR of TccC3 is more similar to *S. typhimurium* SpvB, as it ADP-ribosylates actin directly (Aktories *et al.*, 2012 ▸), although TccC3 functions by stabilizing filamentous actin, whereas SpvB modifies monomeric actin.

The *C. botulinum* C3-like ADP-ribosyltransferase toxins recognize Rho as their substrate via a conserved, solvent-exposed phenyl­alanine residue (F208 in C3 toxin) in a loop of the central β-sheet of the enzyme. The loop (indicated by a red arrow in Fig. 4 ▸) reaches across the active site to interact with the helical domain, supported by the N-terminal α-helix. During substrate recognition, the exposed phenyl­alanine recognizes a hydro­phobic pocket on the surface of Rho, and positions a conserved glutamine residue (Q211 in C3 toxin) to interact with the residue in Rho (N41) that is the target for ADP-ribosylation (Han *et al.*, 2001 ▸). Neither F208 nor Q211 is conserved in the YenC3^CTD^ sequence, and neither the arrangement of the loop nor the N-terminal helix are conserved in the YenC3^CTD^ structure. This makes it unlikely that Rho is the cellular target for YenC3^CTD^.

In contrast, there are several features of the YenC3^CTD^ structure that are similar to *S. typhimurium* SpvB, including the lack of an N-terminal helix, the presence of an extra helix in the helical domain and a characteristic helical insertion in a loop of the central β-sheet (indicated by a blue arrow in Fig. 4 ▸). We therefore predict from structural similarity that YenC3^CTD^ is most likely an ADP-ribosyltransferase toxin, with actin as its likely cellular target. SpvB is a member of a large family of ADP-ribosyltransferases that modify R177 of actin, consequently inhibiting its polymerization by introducing a steric clash that prevents actin monomers interacting to form a normal filament (Margarit *et al.*, 2006 ▸). This disruption of actin filament formation then leads to an apoptotic mechanism of cell death (Browne *et al.*, 2002 ▸).

### 2.5. Toxin expression profiles in *Y. entomophaga*

**Figure 5:**
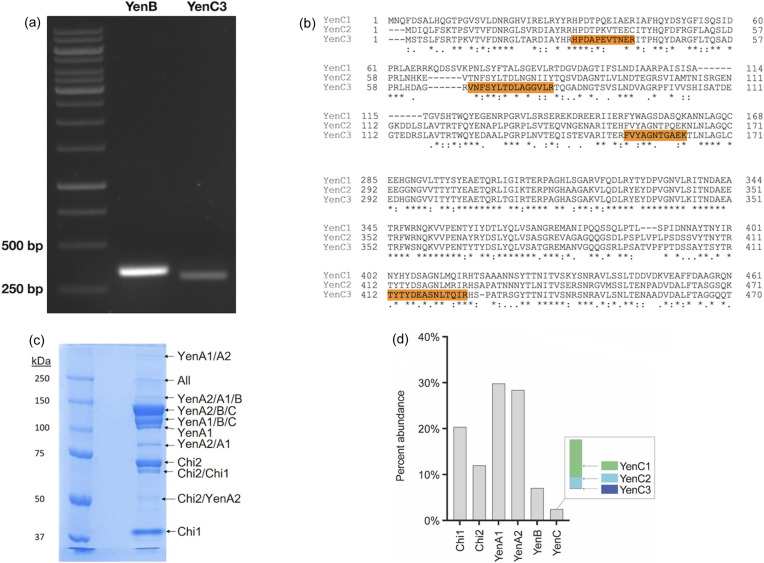
(*a*) RT-PCR of *Y. entomophaga* culture at the late log phase, showing contemporaneous amplification of both *yenB* and *yenC3* genes. Amplicons were visualized on a 1% agarose gel alongside the GeneRuler 1 kb DNA ladder (Thermo Scientific). (*b*) Multiple sequence alignment of YenC1, YenC2 and YenC3, highlighting the diagnostic sequences used to establish the presence of YenC3. (*c*) SDS–PAGE of purified Yen–Tc used to identify the constituent protein subunits. (*d*) Relative abundance of Yen–Tc component proteins, calculated using the summed intensities of unique peptides per protein.

Having established that YenC3 is able to form a complex with YenB *in vitro*, we investigated whether YenB and YenC3 were co-expressed and/or formed functional complexes that could be detected *in vivo*. We first assessed whether mRNA transcripts from both the *yenC3* and the *yenB* genes were present under the same growth conditions. RT-PCR was carried out using gene-specific primers and mRNA prepared from a *Y. entomophaga* culture at the late log phase of growth, a growth phase at which the expression of Yen–Tc had previously been observed (Hurst *et al.*, 2011*b* ▸). We observed the presence of both *yenB* and *yenC3* transcripts [Fig. 5 ▸(*a*)], demonstrating that the genes are contemporaneously expressed, and that YenC3 and YenB are potentially available to combine to form a mature Yen–Tc complex within the cell.

It is known that genetic loci that encode multiple TcC-like proteins give rise to mixed toxin populations containing the same A and B subunits, but combined with different C sub­units (Hurst *et al.*, 2011*b* ▸). To confirm that proteins derived from the detected transcripts were indeed able to form detectable complexes *in vivo*, we carried out mass spectrometry analysis of Yen–Tc purified from *Y. entomophaga* culture supernatant [Fig. 5 ▸(*c*)]. Validating the presence of a subpopulation of Yen–Tc complexes that incorporated YenC3 was challenging due to the high sequence identity between YenC3 and the two known TcC proteins derived from the Yen–Tc pathogenicity island, YenC1 and YenC2 [Fig. 5 ▸(*b*)]. Regardless, we found clear and unambiguous evidence for the presence of four peptide sequences unique to YenC3 in our mass spectrometry data, confirming that YenC3 is indeed secreted as part of a complete Yen–Tc holotoxin assembly, albeit at a much lower level than YenC1 and YenC2. A label-free analysis using the summed intensities of all the unique peptides identified by mass spectrometry across all the gel bands analysed suggested that YenC3 is the least abundant of the three TcC proteins present in Yen–Tc, with a relative abundance of approximately less than 1% [Fig. 5 ▸(*d*)]. We therefore conclude that YenC3 is a *bona fide* Yen–Tc subunit, despite the fact that the gene coding for it is located a considerable distance from the *Y. entomophaga* pathogenicity island that encodes the cognate components required for it to form a functional, secreted toxin complex, and is a minor, but nonetheless potent cytotoxic component of the mature Yen–Tc holotoxin population.

## 3. Discussion

*Y. entomophaga* has a single characterized toxin complex locus containing two open reading frames encoding the TcA protein, a single *tcB* gene, two *tcC* genes and two chitinase-encoding genes (Hurst *et al.*, 2011*b* ▸). This work describes the identification of an orphan *tcC* gene at a different locus in the *Y. entomophaga* genome. The YenC3 protein produced from this orphan gene is able to form a complex with YenB, encapsulate its C-terminal cargo and perform the self-cleavage necessary to enable subsequent delivery when incorporated into a functional holotoxin assembly.

Organisms containing polymorphic toxin systems (Zhang *et al.*, 2012 ▸; Jamet & Nassif, 2015 ▸) often have orphan modules (incomplete gene fragments consisting of the C-terminal toxin-containing portion) at other locations in their genome, allowing them to rapidly generate different toxin complexes by genetic rearrangement. In this case, however, the orphan *tcC* gene (*yenC3*) is expressed at the same time as *yenB*, suggesting that it forms a complex with the TcA and TcB components during assembly of the complete ABC toxin complex. This would lead to a mixed population of toxin complexes comprising at least three different toxic payloads, and presumably increasing its overall observed potency. This is in agreement with our previous work, showing that individual TcC proteins are unable to cause the wide variety of cellular effects seen with the natively produced toxin complex (Marshall *et al.*, 2012 ▸). Bioinformatic analysis of YenC1^CTD^ suggests that it contains a cytotoxic necrotizing factor domain (Hurst *et al.*, 2011*b* ▸), which would modify Rho. YenC2^CTD^, on the other hand, contains a predicted nucleic acid deaminase (Busby *et al.*, 2013 ▸) and presumably causes cell death by DNA and/or RNA damage rather than disruption of the cyto­skeleton. We speculate that the presence of a third *tcC* gene encoding a third, different C-terminal effector cargo targeting actin may in part explain the high potency of the complete Yen–Tc – it shows an LD_50_ of 1.59 fmol per diamond-back moth larva (Landsberg *et al.*, 2011 ▸).

The crystal structure of YenB/YenC3 shows clear electron density for the majority of YenB and YenC3^NTD^. Electron density for the remaining, otherwise unaccounted for protein was patchy, and despite the fact that a model of the YenC3^CTD^ HVR could not be built into this density, SAXS analysis confirmed that the YenC3^CTD^ is contained within the hollow shell formed by YenB and YenC3^NTD^. The interior surface of this shell is mainly positively charged with hydro­phobic patches, features that likely maintain the cargo in an unfolded state (Busby *et al.*, 2013 ▸; Meusch *et al.*, 2014 ▸). Direct evidence for this was found on closer inspection of the crystal structure, with areas of electron density attributable to YenC3^CTD^ packing against hydro­phobic grooves or stacking on aromatic residues.

The structure of the YenC3^CTD^ HVR shows it adopts a mono-ADP-ribosyltransferase fold, with structural similarities to the *S. typhimurium* virulence protein SpvB, suggesting that the YenC3^CTD^ HVR modifies actin directly. Targeting actin would mean that YenC3^CTD^ is an approximate functional analogue of the HVR of TccC3 from *P. luminescens*, despite the proteins being undetectably similar in amino acid sequence. The structure of TccC3 has recently been determined using solution NMR spectroscopy and its mechanism of action established (Belyy *et al.*, 2022 ▸). Like YenC3^CTD^, TccC3 is a two-domain protein, with an N-terminal helical domain and a C-terminal ADP-ribosyltransferase domain. However, TccC3 has a non-canonical structure, with both domains showing considerable topological deviation from other structurally characterized ADP-ribosyltransferases, and a different substrate-binding mechanism. However, it does retain a functional core NAD^+^-binding region, and exerts cellular toxicity by ADP-ribosylating residue T148 of F-actin, preventing the severing of actin filaments by the actin de­polymerizing factor cofilin.

The N-terminal poly proline repeat region of YenC3^CTD^ is unlikely to play a direct catalytic role in the toxin mechanism. However, it does bear superficial similarity to the formin homology region 1 (FH1) domain (PFAM PF06346). The FH1 domain binds to profilin which in turn localizes it to the fast-growing barbed end of actin filaments (Evangelista *et al.*, 2003 ▸). We speculate that the poly proline region of YenC3^CTD^ may function in a similar manner, helping to localize the toxin to its actin target.

In summary, this study describes the structure and proposed function of a previously uncharacterized toxic payload of the ABC toxin complex from the entomopathogenic bacterium *Y. entomophaga*. This toxin is expressed at the same stage as other components of the ABC complex and can bind to the rest of the toxin complex machinery to reconstitute a full ABC complex. Hence, apparently orphaned toxin genes present throughout bacterial genomes can be incorporated into complete ABC complexes, further expanding the repertoire of toxins that can be delivered by a shared mechanism. This situation may also be the case for other RHS-repeat containing proteins that are found in many bacterial genomes.

## 4. Materials and methods

### 4.1. Cloning, expression and purification

The genes *yenB* and *yenC3* from *Y. entomophaga* (GenBank accession Nos. PL78_03760 and PL78_18780, respectively) were cloned into the pET-Duet1 plasmid for co-expression. *yenC3* ^CTD^ (consisting of residues 740–965) was cloned into the pDEST17 expression plasmid. Expression was performed in ZYM-5052 auto-induction medium (Studier, 2005 ▸), and cell lysis and protein purification were carried out as previously described (Busby *et al.*, 2012 ▸).

### 4.2. Crystallization and structure determination of YenB/YenC3

Initial crystallization screens were performed in 96-well sitting-drop format with protein at 10.6 mg ml^−1^. Fine screening was performed, with the best crystals growing in 22%(*w*/*v*) PEG 6000, 0.2 *M* TAPS pH 8.5. Crystals were cryoprotected by briefly soaking in mother liquor containing 20%(*v*/*v*) glycerol and snap-cooled in liquid nitro­gen. Diffraction images were collected over 472° of rotation to a resolution of 2.4 Å using a Rigaku MicroMax 007HF X-ray generator and a mar345dtb image plate detector. The crystal was maintained at 110 K by a cryostream of cold nitro­gen gas. Data were integrated using *XDS* (Kabsch, 2010 ▸) and scaled and merged using *AIMLESS* (Evans, 2011 ▸). Molecular replacement was performed with *Phaser* (McCoy *et al.*, 2007 ▸), using the structure of YenB/YenC2^NTD^ (PDB entry 4igl) as a model. The structure was refined by rounds of manual model building with *Coot* (Emsley *et al.*, 2010 ▸) and refinement with *REFMAC5* (Murshudov *et al.*, 2011 ▸). Data processing and model statistics are presented in Table 1 ▸, and the structure has been deposited in the Protein Data Bank (PDB entry 5kis).

### 4.3. Crystallization and structure determination of YenC3^CTD^

Crystal screens were performed in 96-well sitting drop format with protein at 17.4 mg ml^−1^. Conditions were fine-screened, with the best being 29%(*w*/*v*) PEG 2000 MME, 0.15 *M* KBr. Crystals were derivatized by transferring into a drop containing 30%(*w*/*v*) PEG 2000 MME, 0.15 *M* KBr, 10 m*M* K_2_PtCl_4_ and incubating for 10 min. Crystals were then briefly back-soaked in 30%(*w*/*v*) PEG 2000 MME, 0.15 *M* KBr, 25%(*v*/*v*) glycerol and snap-cooled in liquid nitro­gen. Diffraction data were collected on the MX2 beamline of the Australian Synchrotron in two positions along the crystal, with 360° collected at each position. Crystals were maintained at 100 K by a stream of cold nitro­gen gas. Diffraction data were collected at the platinum *L*_III_-edge (wavelength 1.07219 Å) with an EIGER X 16M photon counting detector in 0.1° wedges. Data were integrated and scaled using *XDS* (Kabsch, 2010 ▸) and merged using *AIMLESS* (Evans, 2011 ▸). Heavy atom sites were found and SAD phasing was performed using *SHELX* (Sheldrick, 2010 ▸). Density modification was performed with *DM* (Cowtan & Main, 1998 ▸) and this produced electron density sufficient to allow automatic model building with *ARP/wARP* (Langer *et al.*, 2008 ▸). This was followed with rounds of manual model building in *Coot* (Emsley *et al.*, 2010 ▸) and refinement with *REFMAC5* (Murshudov *et al.*, 2011 ▸). Data processing and model statistics are presented in Table 1 ▸ and the structure has been deposited in the PDB (PDB entry 6aqk).

### 4.4. SEC-MALLS

To assess protein homogeneity and oligomeric state, size-exclusion chromatography with multi-angle laser light scattering (SEC-MALLS) was used. A dilution series consisting of 100 µl protein samples at 3.7, 1.8 and 0.9 mg ml^−1^ was used. Samples were run through a Superdex S200 Increase 10/300GL column, and a Dionex HPLC, PSS SLD7000 7-angle MALLS detector and Shodex RI-101 differential refractive index detector were used. Molecular weight calculations were performed using PSS *winGPC Unichrom* software.

### 4.5. SAXS

Protein was purified to homogeneity as determined by SEC-MALLS and SDS–PAGE (Fig. S3). Protein was exhaustively dialysed against 20 m*M* HEPES pH 7.5, 150 m*M* NaCl, 0.5 m*M* TCEP, with a sample of the dialysate used as the solvent blank. Small-angle scattering data were collected for a concentration series at the SAXS/WAXS beamline of the Australian Synchrotron. Data collection and processing statistics are presented in Table 2 ▸ and Fig. S4. Data were integrated using *scatterBrain* (Australian Synchrotron, 2019 ▸) and subsequent analysis performed using the *ATSAS* suite (Petoukhov *et al.*, 2012 ▸). Scattering data were placed on the absolute scale by normalization against water (Orthaber *et al.*, 2000 ▸). Data were extrapolated to zero concentration using *PRIMUS* (Konarev *et al.*, 2003 ▸) prior to *ab initio* modelling.

Model building was performed using 20 runs of *DAMMIF*, followed by model superposition and selection using *DAMSUP*, *DAMSEL*, *DAMAVER*, *DAMSTART* and a final model refinement run with *DAMMIN*. The 20 initial models had a mean NSD of 0.546 and a standard deviation of 0.041; 19 of the models were selected to be used in the averaging process.

### 4.6. Molecular graphics

Figs. 1 ▸–4 ▸ ▸ ▸ were prepared using *The PyMOL Molecular Graphics System*, version 1.8, Schrödinger, LLC (DeLano, 2002 ▸).

### 4.7. Reverse transcriptase PCR

For preparation of *Y. entomophaga* RNA, *Y. entomophaga* was grown in LB media to a cell density of 4.4 × 10^9^ colony forming units ml^−1^. Two 200 µl aliquots were independently aliquoted into 1.5 ml micro centrifuge tubes containing 400 µl of RNAprotect Bacteria Reagent and RNA was then prepared using the RNeasy Mini Kit (Qiagen), following the manufacturer’s instructions. The concentration of the resultant RNA was measured using a Nanodrop ND-1000 spectrophotometer and the integrity was confirmed by 1% agarose gel electrophoresis.

cDNA synthesis was performed using the SuperScript IV First-Strand Synthesis System (Invitrogen). 4 µl of 2.5 µ*M* dNTP mix, 1 µl of 50 µ*M* Random Hexamers (Promega), 0.5 µl template RNA (1474 ng µl^−1^) and 7.5 µl DEPC-treated H_2_O was added and the sample incubated at 65°C for 5 min, followed by 1 min on ice. A solution containing 4 µl 5× Super Script IV Buffer, 1 µl 100 m*M* DTT, 1 µl RNaseOUT (Invitrogen) recombinant RNase inhibitor and 1 µl SuperScript IV Reverse Transcriptase were then added and the sample was mixed and incubated at 23°C for 10 min. The reactions were transferred to 55°C for 15 min, followed by a 10 min heat inactivation at 80°C. The RT control comprised the same protocol as above but with 1 µl DEPC-treated H_2_O used in place of SuperScript IV Reverse Transcriptase.

50 µl PCR reactions were performed using Platinum Taq PCR Kit (Invitrogen). Combined 5 µl 10× PCR Buffer, 2 µl 50 m*M* MgSO_4_, 1 µl 2.5 µ*M* dNTPs (Invitrogen), 0.2 µl Taq DNA polymerase, 0.5 µl each primer (40 µ*M*), 1 µl Template (either cDNA, -RT control or untreated RNA control) and 39.8 µl H_2_O. Primer sequences: YenB-F: 5′-CTC ATC GCG TCC AAT AGC CT-3′, YenB-R: 5′-CGT ACT ACG CTG GCT GAG AG-3′, YenC3-F: 5′-AGC AAC TTG ACC GAA AAC GC-3′, YenC3-R: 5′-GCT TTC CGT TTC CGT ATC GC-3′. PCR cycles as follows: 95°C, followed by 32 cycles (95°C 30 s, 57°C 30 s, 72°C 60 s) with a final 5 min at 72°C.

### 4.8. Mass spectrometry

To assess the presence and abundance of YenC3 in native toxin complexes, native Yen–Tc was purified from the supernatant of *Y. entomophaga* cultures grown in LB broth, essentially as described previously (Jones and Hurst, 2016 ▸). Following Superose 6 SEC, proteins were in-gel digested with trypsin according to Shevchenko *et al.* (2006 ▸) with minor alterations. Liquid chromatography conditions were as described previously (O’Brien *et al.*, 2020 ▸) except that peptides were separated with a gradient of 5 to 50% solvent B over 34 min, then 50–98% B over 6 min. The samples were analysed on an Orbitrap Elite mass spectrometer (ThermoFisher Scientific). Survey scans were acquired in the Orbitrap at an *m*/*z* of 350 to 1800 with a resolution of 60 K using a maximum injection time of 200 ms. The ten most intense precursors with charge states above two were selected for MS2 in the ion trap using an isolation window of 2 Da, a maximum injection time of 150 ms and normalized collision energy of 35%.

### 4.9. Data analysis

The Sequest HT node in *Proteome Discoverer* (version 2.5.0.400; ThermoFisher Scientific) was used to search the combined spectra from all excized gel slices. The protein database contained eight *Y. entomophaga* protein sequences with a custom contaminants database. Cleavage specificity was set as trypsin, enzyme specificity was set as specific and a maximum of two missed cleavages were allowed. Mass tolerances of 15 p.p.m. and 0.6 Da were applied to precursor and fragment ions, respectively. Cys-*S*-β-propionamide was set as a static modification, and dynamic modifications were set to deamidation of Asn and mono-oxidized Met. Confident peptide-to-spectrum matches (PSMs) were assigned using the Percolator node with default settings. For label-free quantification, precursor peak intensities were obtained using the ‘Minora Feature Detector’ node in the processing workflow and the ‘Feature Mapper’ and ‘Precursor Ions Quantification’ nodes in the consensus workflows. Protein abundance was calculated by dividing the summed intensities of unique PSMs for a single protein by the summed intensities of unique PSMs from all *Y. entomophaga* proteins.

## Supplementary Material

Supporting figures. DOI: 10.1107/S2052252524001969/tf5004sup1.pdf

PDB reference: YenB/RHS2 complex, 5kis

PDB reference: the C-terminal toxin domain of RHS2 from *Y. entomophaga*, 6aqk

## Figures and Tables

**Table 1:** Crystallographic data collection and refinement statistics

	YenB/YenC3	YenC3^CTD^
Wavelength (Å)	1.54056	1.07219
Resolution range (Å)	32.51–2.40 (2.44–2.40)	50.41–1.80 (1.85–1.80)
Space group	*C*121	*P*212121
Unit cell		
*a*, *b*, *c* (Å)	148.61, 132.76, 155.36	37.51, 54.52, 100.82
α, β, γ (°)	90, 103.85, 90	90, 90, 90
Total reflections	1141459 (55264)	499693 (25906)†
Unique reflections	113222 (5510)	36952 (2674)†
Multiplicity	10.1 (10.0)	13.5 (9.7)
Completeness (%)	99.2 (98.2)	99.8 (97.7)
Mean *I*/σ*I*	9.4 (0.6)	11.92 (1.00)
Wilson *B* factor (Å^2^)	40.8	42.03
*R*_meas_	0.305 (4.434)	0.111 (2.212)
CC_1/2_	0.990 (0.206)	0.999 (0.399)
*R*_work_	0.216	0.181
*R*_free_	0.260	0.226
No. of atoms	16843	1804
No. of macromolecules	16616	1711
No. of ions	8	16
No. of waters	219	77
RMSD angles	1.305	1.824
RMSD bonds	0.009	0.0185
Ramachandran statistics		
Favoured (%)	97.1	98.6
Allowed (%)	2.8	1.4
Outliers (%)	0.1	0
Clashscore	1	1.8

† Friedel pairs treated as different reflections.

**Table 2:** SAXS data collection statistics

Data collection parameters	
Instrument	SAXS/WAXS beamline at the Australian Synchrotron
Detector	Pilatus 1M
Beam geometry	point
Wavelength (Å)	1.0332
Camera length (mm)	3252
*q* range (Å^−1^)	0.005-0.292
Exposure time (s)	1
Concentration range (mg ml^−1^)	0.15–2.4
Temperature (K)	285

Structural parameters†	
*I*(0) (cm^−1^) [from *P*(*r*)]	0.48
*R*_g_ (Å) [from *P*(*r*)]	43.99
*I*(0) (cm^−1^) [from Guinier]	0.48
*R*_g_ (Å) [from Guinier]	43.85
*D*_max_ (Å)	153.06
Calculated molecular mass‡ [from *I*(0)] (kDa)	276.9
Calculated molecular mass (from SAXS MoW2) (kDa)	269.0
Molecular mass from sequence (kDa)	273.8

Software employed	
Primary data reduction	*scatterBrain*
Data processing	*PRIMUS*
*Ab initio* analysis	*DAMMIF*/*DAMMIN*
Validation and averaging	*DAMAVER*
Computation of model intensities	*CRYSOL* §
Three-dimensional graphics representation	*PyMOL*

† Calculated for a 2.4 mg ml^−1^ concentration.

‡ Calculated from data placed on an absolute scale and using a partial specific volume of 0.7425 cm^3^ g^−1^.

§ Svergun *et al.* (1995 ▸).
