# Supplementary material for: The ABC toxin complex from Yersinia entomophaga can package three different cytotoxic components expressed from distinct genetic loci in an unfolded state: the structures of both shell and cargo
Source: IUCrJ. 2024 May 1;11(Pt 3):299–308. doi: 10.1107/S2052252524001969 (PMC11067744; doi:10.1107/S2052252524001969)

IUCrJ

Volume 11 (2024)

Supporting information for article:

The ABC toxin complex from *Yersinia entomophaga* can package three different cytotoxic components expressed from distinct genetic loci in an unfolded state: the structures of both shell and cargo

Jason N. Busby, Sarah Trevelyan, Cassandra L. Pegg, Edward D. Kerr, Benjamin L. Schulz, Irene Chassagnon, Michael J. Landsberg, Mitchell K. Weston, Mark R. H. Hurst and J. Shaun Lott

Supplementary Data 1: Multiple sequence alignment of the three TcC subunits of Yen-Tc

```
YenC1 : MNQFDSALHQGTPGVSVLDNRGHVIRELRYYRHPDTPQEIAERIAFHQYDSYGFISQSIDPRLAERRKQDSSVKPNLSYFTALSGEVLRT :  90
YenC2 : ---MDIQLFSKTPSVTVFDNRGLSVRDIAYRRHPDTPKVTEECITYHQFDFRGFLAQSLDPRLNHKE------VTNFSYLTDLNGNIIYT :  81
YenC3 : ---MSTSLFSRTPKVTVFDNRGLTARDIAYHRHPDAPEVTNERITPHQYDARGFLTQSADPRLHDAG------RVNFSYLTDLAGGVLRT :  81

YenC1 : DGVDAGTIFSLNDIAARPAISISA------------TGVSHTWQYEGENRPGRVLSRSEREKDREERIIERFYWAGSDASQKANNLAGQC : 168
YenC2 : QSVDAGNTLVLNDTEGRSVIAMTNISRGENGKDDLSLAVTRTFQYENAPLPGRPLSVTEQVNGENARITEHFVYAGNTPQEKNLNLAGQC : 171
YenC3 : QGADNGTSVSLNDVAGRPFIVVSHISATDEGTEDRSLAVTRTWQYEDAALPGRPLNVTEQISTEVARITERFVYAGNTGAEKTLNLAGLC : 171

YenC1 : LRHYNSAGLNQTLSIALTGTPISACFQPLLESA----EPEWQGTNESAWLELLTPEIFTTYNRADANGETLVQTDAMGNIQRLAYDVAGF : 254
YenC2 : VSYYDAAGLIQTDSVSLTGKPLSVSRKLLKNLDDTNILADWQGNDTSAWNSLLATEIYTTVTRTDAAGAVLTTIDAVGNQQRVAFDIAGQ : 261
YenC3 : VRHYDTAGLVQTDSIALTGVSLSVTRRLLKDADNPDTVADWQGEGASAWNDLLSGEEYVTLTTADATGTVLTTTDAKGNIQRVRYDVAGL : 261

YenC1 : LKSSWLSLKGGQEQIIVKSLTYSAAGQKLQEEHGNGVLTTYSYEAETQRLIGIRTERPAGHLSGARVFQDLRYTYDPVGNVLRITNDAEA : 344
YenC2 : LSASWLTLKGGQEQVIIKVLTYSAAGQKLREEGGNGVVTTYTYEAETQRLIGIKTERPNGHAAGAKVLQDLRYEYDPVGNVLSITNDAEE : 351
YenC3 : LSGSWLTVRDRTEQVIVKSLTYSAAGQKQREDHGNGVVITYTYEAETQRLTGIRTERPAGHASGAKVLQDLRYEYDPVGNVLKITNDAEA : 351

YenC1 : TRFWRNQKVVPENTYIYDTLYQLVSANGREMANIPQQSSQLPTL---SPIDNNAYTNYIRNYHYDSAGNLMQIRHTSAAANNSYTTNITV : 431
YenC2 : TRFWRNQKVVPENAYRYDSLYQLVSASGREVAGAGQQGSDLPSPLVPLPSDSSVYTNYTRTYTYDSAGNLMRIRHSAPATNNNYTLNITV : 441
YenC3 : TRFWSNQKVVPENTYTYDSLYQLVSATGREMANVGQQGSRLPSATVPFPTDSSAYTSYTRTYTYDEASNLTQIRHS-PATRSGYTTNITV : 440

YenC1 : SKYSNRAVLSSLTDDVDKVEAFFDAAGRQNQLLPGQTLSWNARGELAKVTPVARDGQESDSETYRYDANSQRVSKMAIQQSNNNTQTRRV : 521
YenC2 : SERSNRGVMSSLTENPADVDALFTASGSQKCLQQGQSLIWTPRGELRTVLLVARGETADDSESYRYDGSSQRILKISSQQTNHSARVQRA : 531
YenC3 : SNRSNRAVLSNLTENAADVDALFTAGGQQTQLQPGLGLVWTARNELLKVTPVMRDGSADDSENYRYDGGSQRILKVSVQKTGNSAQTQRA : 530

YenC1 : LYLAGLERRTIHQGNTLFETLLVVKIGEAGRAQVQAMHWELGQPTEVANDELRYSYDNLIGSSGLEVDGTGQLISQEEYYPYGGTAVWMA : 611
YenC2 : LYLPGLEWRTMTGGVAEAENLQVICIGEAGRAQVRVLHWESGKPDGIINDQIRWSYDNLTCSSGLEVDGDGLVISMEEYYPYGGTAVWAA : 621
YenC3 : LYLPGLELRSAKNGDTETESLQVITVGEASRAQVRMLHWESGRPDGITDDKVRYSYDNLTGSSVLELDSDGKLISMEEYYPYGGTAVWTV : 620

YenC1 : RSQREASDKAYGYSGKERDATGLYYYGFRYYQPWAGRWLSADPAGTIDGLNLFRMVRNNPIVLHDPDGLAPGFFERISSFRKKDTLTISS : 701
YenC2 : RSHIETAYKTVRYSGKERDATGLYYYGFRYYQPWAGRWLSADPAGTVDGLNLYRMVRNNPLRLTDPDGMAPLDWLDLDTTNASRDI---- : 707
YenC3 : RSAVEANYKTVRYSGKERDATGLYYYGYRYYQPWAGRWLSADPAGSVDGLNLYRMVRNNPVAWKDNDGRIPINTMIPPPPPMMGGN---- : 706
                                    *            *                      *  *^

YenC1 : LKGTGPFYTRSESEIDIDFLFSRQDRDKDFPPQNHKE-LSAEDRREVLEVSSGENITSA--------------------NKSSKWYAGTH : 770
YenC2 : VK---AIYQLN---------------------QIDGPHRGVRDTYQRMTESTGMILQETLNNEAVLKGIKQKDKEKKSRGMKFT------ : 767
YenC3 : PPPPPPMMGGNPPPPP-----PVAGGNPPPPPSMPGQQNGAKKNWV---------I-------------KEDPALYKQQGGEYPYYSSFT : 769

YenC1 : WETK--PLKNNTDLVVLHNGVQGAAGININLNDIKPGRSV------------LVTAGTLTGCTMITGVKGNNFYALHAGTGTPSENWVTG : 846
YenC2 : ----NSKLKTYAAHAGVL-------------NTL--QPDPVYKDGFLNLPGSLGNKNTFPGVELIEDKVKPSLSQYHPDKLGKSQRWK-P : 837
YenC3 : IALQKLNISHYDSIIDMDNFISKWAEIG---RNMKPAARDISHDKFIEVQKTLG-------------KIDAEWSGYHSADVNETFRGDTP : 843

YenC1 : EHGVTDNFRMLNKLIPDAGIDLNPEAVNDSLLTILDYFDNGT-IAYNGKKGSEIHRDADNILNYRTTGYENTVGVSFSLLTKDKNGEVSA : 935
YenC2 : ESSLG--YY---------------------RVADTEAFITGIRSQYK-SSGTDLHAVVEGRIRDHLLAN-NNVLPKMAGI-AGLHAEVQA : 901
YenC3 : VISNS--YS---------------------WLA---EFINES----E-GKSDTVQKSMDLEIKSPLIMS-TAKDPKMGYV-SGKT----- : 895

YenC1 : S-TLLELGELKPHKKHRTRGQFGMT------------------------------------ELKYEARKNTVVKLR----- : 974
YenC2 : LNYIISNPDIEGGNAERLNGSYIFTQRLVGDVNQDFPACYNCSGIISGLENVMTGRVNNDV--RLKRRKSF---------- : 970
YenC3 : ---IMWHFDLEPGHAGVSEGLYASEGE------VTFPL-YNRMK-ITSLQYLPEGRSYMDNPEQYGTSHRYIIKARMLPRS : 965
```

Supplementary Data 2: SAXS fitting of YenB/YenC3^{NTD} and YenC3^{CTD}

To provide further support for the idea that YenC3^{CTD} is encapsulated within the YenB/YenC3^{NTD} shell, further analysis was performed using the SAXS data collected previously. The crystal structures of YenB/YenC3^{NTD} and YenC3^{CTD} were positioned relative to each other by fitting to the scattering curve of the YenB/YenC3^{NTD}/YenC3^{CTD} complex using SASREF (Petoukhov, M. V. & Svergun, D. I. (2005) Global rigid body modeling of macromolecular complexes against small-angle scattering data. *Biophys J* **89**, 1237-1250 https://doi.org/10.1529/biophysj.105.064154). This process placed the YenC3^{CTD} structure inside the hollow cavity of YenB/YenC3^{NTD} (Panel A), and the calculated scattering from this model provided a better match to the experimental data than the scattering data from YenB/YenC3^{NTD} alone (Panels B and C). The model scattering does not perfectly match the experimental scattering, likely due to the cargo protein being in an unfolded, disordered state. These results provide direct experimental support to confirm that the C-terminal "cargo" of ABC toxins is indeed encapsulated inside the hollow shell formed by the TcB/TcC^{NTD} protein complex.

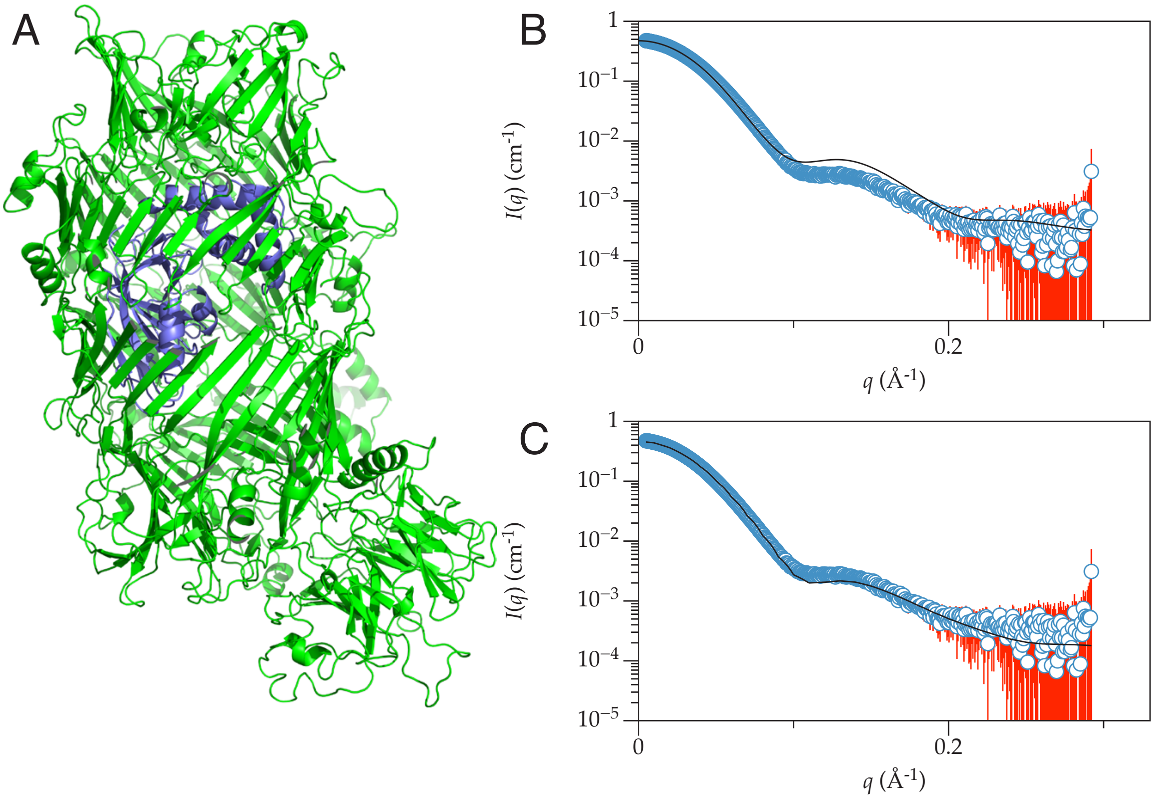

A Fitting of the YenB/YenC3^{NTD} and YenC3^{CTD} crystal structures resulted in the YenC3^{CTD} structure being placed within the cavity of the YenB/YenC3^{NTD} structure.

B The theoretical scattering of the YenB/YenC3^{NTD} model (black line) and its fit to the experimental scattering of YenB/YenC3^{NTD}/YenC3^{CTD}. $\chi^2 = 20.083$.

C The theoretical scattering of the complex shown in **A** (black line) and its fit to the experimental small-angle X-ray scattering of YenB/YenC3^{NTD}/YenC3^{CTD} (blue circles). Error bars (2 σ) are shown in red. $\chi^2 = 7.75$.

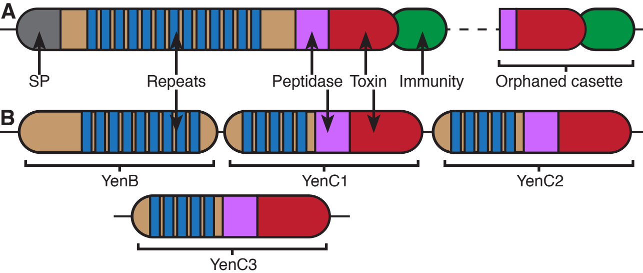
A
SP
Repeats
Peptidase
Toxin
Immunity
Orphaned casette
B
YenB
YenC1
YenC2
YenC3

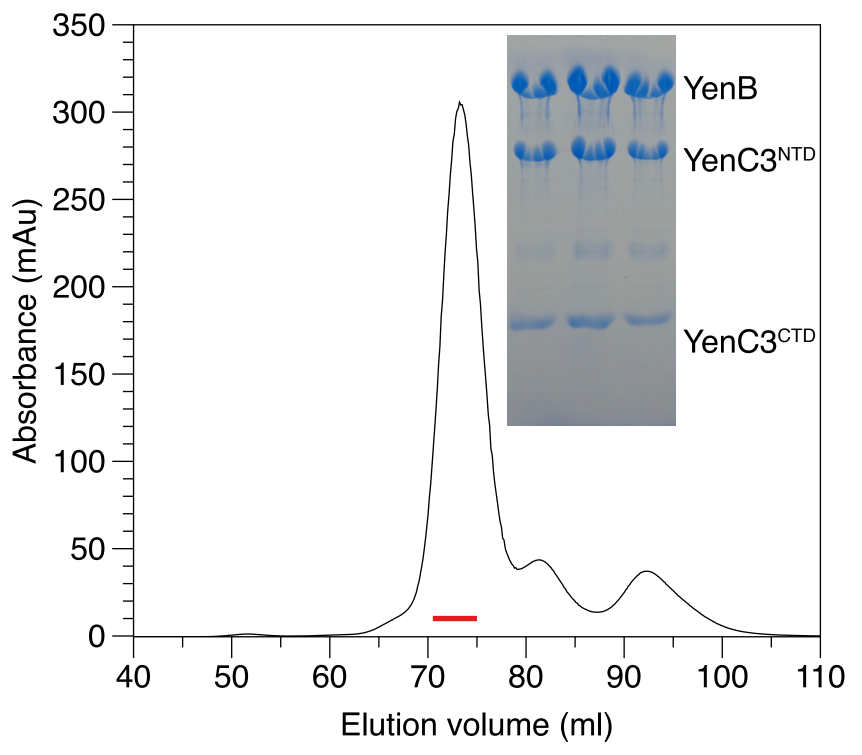

YenB
YenC3^{NTD}
YenC3^{CTD}
Absorbance (mAu)
Elution volume (ml)
0
50
100
150
200
250
300
350
40
50
60
70
80
90
100
110

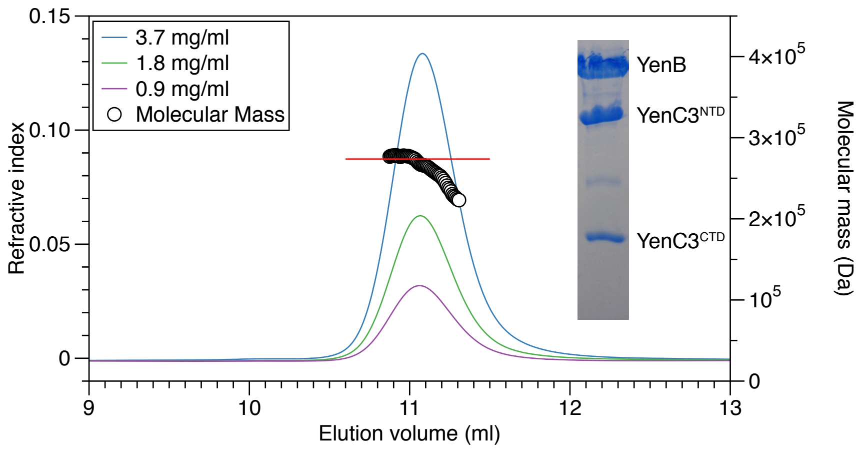

0.15
0.10
0.05
0
Refractive index
3.7 mg/ml
1.8 mg/ml
0.9 mg/ml
Molecular Mass
YenB
YenC3NTD
YenC3CTD
4×10^5
3×10^5
2×10^5
10^5
0
Molecular mass (Da)
9
10
11
12
13
Elution volume (ml)

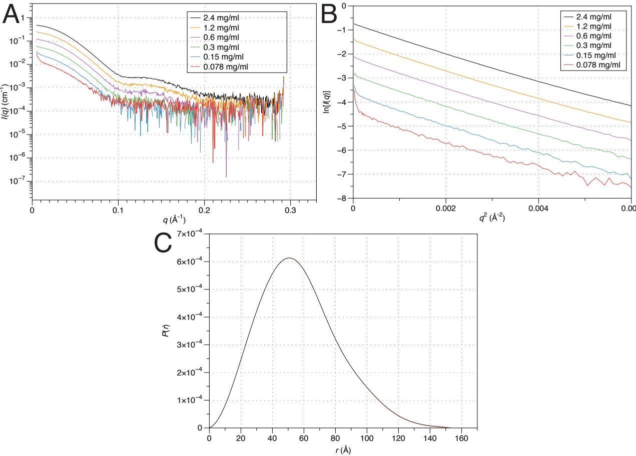

A
B
C
2.4 mg/ml
1.2 mg/ml
0.6 mg/ml
0.3 mg/ml
0.15 mg/ml
0.078 mg/ml
I(q) (cm⁻¹)
q (Å⁻¹)
ln[I(q)]
q² (Å⁻²)
P(r)
r (Å)

Supplementary Figure 5. Annotated spectra of the four unique peptides observed for YenC3. The highest scoring peptide-to-spectrum matches (PSMs) were used and all spectra were manually validated.

i. HPDAPEVTNER

#1	b^+	b^{2+}	b^{3+}	Seq.	y^+	y^{2+}	y^{3+}	#2
1	138.06619	69.53673	46.69358	H				11
2	235.11895	118.06311	79.04450	P	1127.53274	564.27001	376.51577	10
3	350.14590	175.57659	117.38682	D	1030.47998	515.74363	344.16484	9
4	421.18301	211.09514	141.06585	A	915.45304	458.23016	305.82253	8
5	518.23577	259.62152	173.41678	P	844.41592	422.71160	282.14349	7
6	647.27837	324.14282	216.43097	E	747.36316	374.18522	249.79257	6
7	746.34678	373.67703	249.45378	V	618.32056	309.66392	206.77837	5
8	847.39446	424.20087	283.13634	T	519.25215	260.12971	173.75557	4
9	961.43739	481.22233	321.15065	N	418.20447	209.60587	140.07301	3
10	1090.47998	545.74363	364.16484	E	304.16155	152.58441	102.05870	2
11				R	175.11895	88.06311	59.04450	1

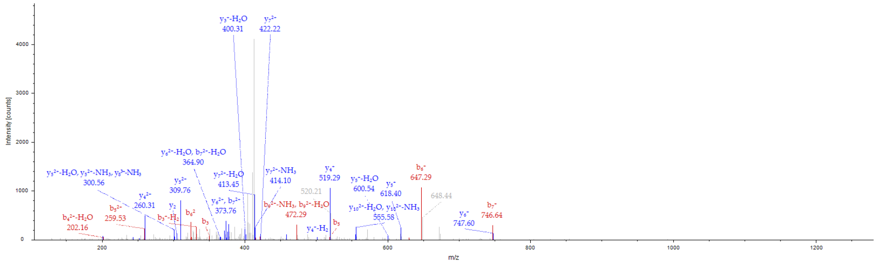

ii. VNFSYLTDLAGGVLR

#1	b^+	b^{2+}	Seq.	y^+	y^{2+}	#2
1	100.07569	50.54148	V			15
2	214.11862	107.56295	N	1525.80092	763.40410	14
3	361.18703	181.09715	F	1411.75799	706.38263	13
4	448.21906	224.61317	S	1264.68958	632.84843	12
5	611.28239	306.14483	Y	1177.65755	589.33241	11
6	724.36645	362.68686	L	1014.59422	507.80075	10
7	825.41413	413.21070	T	901.51016	451.25872	9
8	940.44107	470.72418	D	800.46248	400.73488	8
9	1053.52514	527.26621	L	685.43554	343.22141	7
10	1124.56225	562.78476	A	572.35147	286.67937	6
11	1181.58372	591.29550	G	501.31436	251.16082	5
12	1238.60518	619.80623	G	444.29289	222.65009	4
13	1337.67359	669.34043	V	387.27143	194.13935	3
14	1450.75766	725.88247	L	288.20302	144.60515	2
15			R	175.11895	88.06311	1

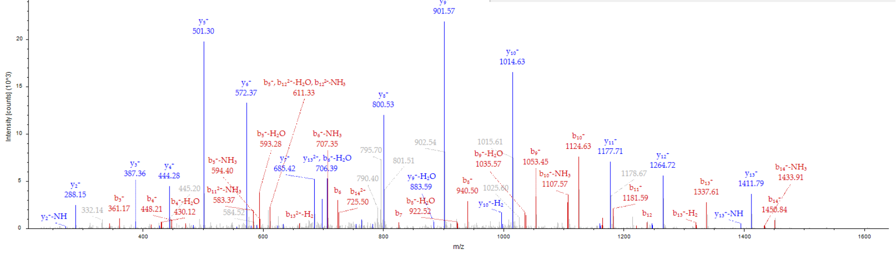

iii. FVYAGNTGAEK

#1	b^+	b^{2+}	Seq.	y^+	y^{2+}	#2
1	148.07569	74.54148	F			11
2	247.14410	124.07569	V	1009.49490	505.25109	10
3	410.20743	205.60735	Y	910.42649	455.71688	9
4	481.24455	241.12591	A	747.36316	374.18522	8
5	538.26601	269.63664	G	676.32604	338.66666	7
6	652.30894	326.65811	N	619.30458	310.15593	6
7	753.35662	377.18195	T	505.26165	253.13446	5
8	810.37808	405.69268	G	404.21397	202.61063	4
9	881.41519	441.21124	A	347.19251	174.09989	3
10	1010.45779	505.73253	E	276.15540	138.58134	2
11			K	147.11280	74.06004	1

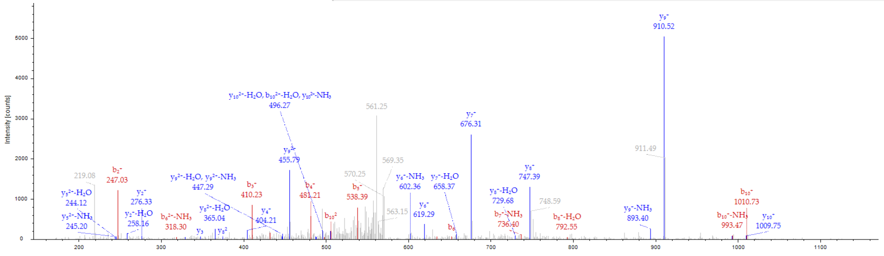

iv. TYTYDEASNLTQIR

#1	b^+	b^{2+}	Seq.	y^+	y^{2+}	#2
1	102.05496	51.53112	T			14
2	265.11828	133.06278	Y	1574.73329	787.87028	13
3	366.16596	183.58662	T	1411.66996	706.33862	12
4	529.22929	265.11828	Y	1310.62229	655.81478	11
5	644.25623	322.63175	D	1147.55896	574.28312	10
6	773.29883	387.15305	E	1032.53201	516.76965	9
7	844.33594	422.67161	A	903.48942	452.24835	8
8	931.36797	466.18762	S	832.45231	416.72979	7
9	1046.39491	523.70109	N-Deamid...	745.42028	373.21378	6
10	1159.47898	580.24313	L	630.39334	315.70031	5
11	1260.52665	630.76697	T	517.30927	259.15827	4
12	1388.58523	694.79625	Q	416.26159	208.63444	3
13	1501.66930	751.33829	I	288.20302	144.60515	2
14			R	175.11895	88.06311	1

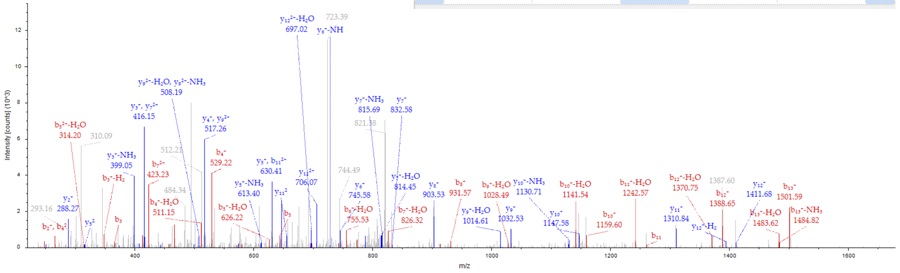

Supplement: Supplementary file 1 [file m-11-00299-sup1.pdf]
